# Residual-based multivariate exponentially weighted moving average control chart for statistical process control of water quality in Surabaya city utilizing generative adversarial network

**DOI:** 10.1016/j.mex.2025.103504

**Published:** 2025-07-12

**Authors:** Muhammad Ahsan, Raditya Widi Indarsanto, Kevin Agung Fernanda Rifki, Muhammad Hisyam Lee

**Affiliations:** a Department of Statistics, Institut Teknologi Sepuluh Nopember, Surabaya, Indonesia; b Department of Data Science and Analytics, Institut Teknologi Kalimantan, Balikpapan, Indonesia; c Department of Mathematical Sciences, Universiti Teknologi Malaysia, Johor Bahru, Malaysia

**Keywords:** Forecasting, Generative adversarial network, Control Chart, MEWMA

## Abstract

This study proposes novel framework to enhance statistical process control (SPC) of water quality by addressing the pervasive issue of autocorrelation in time-series data. We investigate the characteristics of pH, turbidity, and KMnO₄ in Surabaya city's water, revealing significant autocorrelation that compromises statistical independence assumption crucial for reliable SPC. To overcome this, Generative Adversarial Network (GAN) model was developed to generate decorrelated residual time-series. The efficacy of GAN model in reducing autocorrelation was quantitatively validated, achieving Mean Squared Error (MSE) of 0.0054, Root Mean Squared Error (RMSE) of 0.0738, and Mean Absolute Error (MAE) of 0.0556. Subsequently, these GAN-derived residuals were integrated into Multivariate Exponentially Weighted Moving Average (MEWMA) control chart for process monitoring. Phase I analysis detected 33 out-of-control signals; after identifying and removing outliers, process was brought under statistical control with no further out-of-control signals detected. However, subsequent Phase II online monitoring detected eight statistically significant out-of-control signals, indicating a potential loss of process stability over time. Our findings underscore the significant utility of GAN-based residual analysis as a robust strategy for mitigating autocorrelation effects in environmental water quality data. This approach leads to improved process monitoring and enables early anomaly detection, crucial for proactive water quality management.

## Graphical abstract

**Figure:**
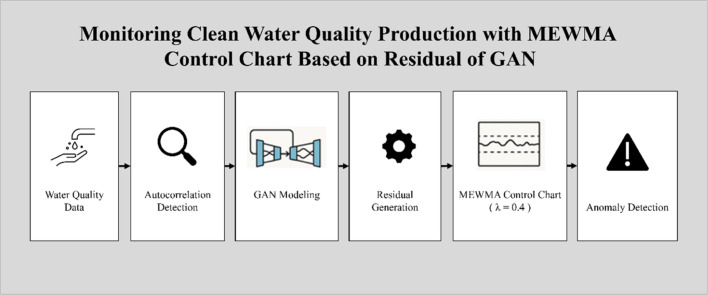

**Key points**

 

• GAN-derived residual analysis shows potential for addressing autocorrelation in water quality monitoring datasets.
• Phase II MEWMA monitoring of GAN residuals revealed process instability despite initial controlled conditions

Alt-text: Unlabelled box

**Specifications table**

**Table:** 

**Subject area**	Mathematics and Statistics
**More specific subject area**	1. Water Quality Monitoring 2. Environmental Data Analysis 3. Time Series Analysis with Machine Learning 4. Statistical Process Control (SPC)
**Name of your method**	**GAN-MEWMA** from: 1. Generative Adversarial Network (**GAN**) – for residual generation 2. Multivariate Exponentionally Weighted Moving Average (**MEWMA**) Control Chart – for statistical monitoring
**Name and reference of original method**	**1. GAN** Ian Goodfellow et al., 2014 **Paper Title**: Generative Adversarial Nets **Reference**: Goodfellow, I., Pouget-Abadie, J., Mirza, M., Xu, B., Warde-Farley, D., Ozair, S., … & Bengio, Y. (2014). Generative adversarial nets. Advances in Neural Information Processing Systems, 27. GAN is used to generate residual values that reduce autocorrelation in water quality data. **2. MEWMA Control Chat** Robert W. Testik & Douglas C. Montgomery Lowry, C.A., Woodall, W.H., Champ, C.W., Rigdon, S.E. **Reference**: Lowry, C. A., & Montgomery, D. C. (1995). A review of multivariate control charts. IIE Transactions, 27(6), 800–810. Lowry, C.A., Woodall, W.H., Champ, C.W., Rigdon, S.E.: A multivariate exponentially weighted moving average control chart. Technometrics. 34, 46–53 (1992). Used for monitoring autocorrelation-adjusted residuals to detect out-of-control signals over time.
**Resource availability**	**Data Source**: pH, turbidity, and KMnO₄ data from laboratory in Surabaya, Indonesia **Modeling Environment**: Python and R Language Studio softwate Computer Hardware

## Background

Surabaya, Indonesia, faces a growing challenge in ensuring sustainable clean water access for its expanding metropolitan population. Water is a critical resource, not just for basic needs but also for public health, economic activity, and urban development. Despite efforts by Surya Sembada, localized water scarcity persists in specific areas, including Wonokromo, Wonokusumo, Wonosari, and parts of North Surabaya. The water treatment process by Surya Sembada water treatment plant in Surabaya City is currently carried out at Ngagel and Karang Pilang installations. However, in its water treatment activities, Surya Sembada water treatment has not been able to implement quality control over the water treatment process using statistical methods. Therefore, this study proposes a multivariate control chart, which handles multivariate data problems using residuals from time series-based machine learning methods [1,2]. This approach involves predicting the water production data, assessing the residuals for autocorrelation, and addressing potential problems [3,4]. A variety of statistical and machine learning techniques can be employed to address this issue. Traditional time series methods such as Vector Autoregression (VAR) models [5], as well as advanced machine learning algorithms including Artificial Neural Networks (ANN) [6,7], Multioutput Least Squares Support Vector Regression (MLS-SVR) [8], XGBoost [9], and Long Short-Term Memory (LSTM) [10,11], offer potential solutions. To improve forecasting accuracy, this study explores the Generative Adversarial Network (GAN) method, which is known for generating realistic synthetic data [12,13]. The application of GAN in water quality forecasting, particularly in water production, offers the advantages of generating accurate synthetic or predicted data and overcoming data limitations.

This study proposes a novel framework that synergizes Generative Adversarial Networks (GANs) with Multivariate Exponentially Weighted Moving Average (MEWMA) control charts to enhance water quality monitoring and operational efficiency in water production. By effectively addressing autocorrelation inherent in water quality data, the GAN-MEWMA model aims to revolutionize traditional monitoring systems. The fidelity of GAN-generated data is paramount in ensuring the accuracy and comprehensiveness of the overall monitoring process. This study seeks to demonstrate the potential of artificial intelligence in improving forecasting capabilities and enabling proactive responses to water quality fluctuations. Ultimately, the integration of this advanced monitoring system is expected to optimize water treatment operations by facilitating timely interventions and preventing quality deterioration.

## Method details

### Statistical quality control and control chart

Statistical quality control (SQC) is a structured methodology employed to attain and maintain desired quality levels within a product or process by systematically reducing process variation [14] It constitutes a statistical framework for the continuous monitoring, measurement, and enhancement of quality attributes [2]. Through the application of statistical tools such as control charts, hypothesis testing, and regression analysis, SQC enables the identification, quantification, and mitigation of process variability. By detecting anomalies, predicting trends, and implementing corrective actions in a timely manner, SQC optimizes operational efficiency, minimizes waste, and elevates customer satisfaction through continual quality improvement. Control charts are indispensable statistical process control tools employed in quality management to visually monitor process variation and identify anomalous shifts or trends [7]. By graphically representing process data relative to established control limits, these charts facilitate the maintenance of acceptable quality levels through the detection of special cause variation [15].

### Generative adversarial network test

Generative Adversarial Networks (GANs) are unsupervised learning models that generate indistinguishable synthetic data. Comprising of two components, the generator and discriminator [16]. The generator is tasked with creating synthetic data that is like original data, where previously generated data was from actual data, while the discriminator tries to differentiate between real or fake data and data generated by the generator. GANs iteratively improve their performance in data creation and differentiation. Forecasting process with a Generative Adversarial Network (GAN) involves a complex series of steps to produce synthetic data. This process starts from collecting actual data as the input vector to producing synthetic data which can be depicted in the diagram below.

Fig. 1

**Fig. 1:**
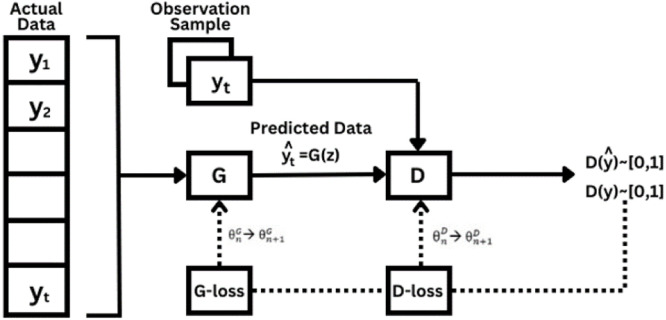
Generative Adversarial Network Forecasting Process.

In the GAN model, synthetic data is produced from random or actual data. This is done by the generator, which uses an Artificial Neural Network (ANN) for processing. In time series forecasting, the generator applies mathematical transformations, using a weight and bias matrix for each layer and an activation function for each neuron’s output to generate synthetic data.

(1) $${\hat{y}}_{t}=G\left(y_{t}\right)$$

(2) $$G\left(y_{t}\right)=\sigma \left(h_{\text{out}}\right)$$

(3) $$h_{\text{out}}=w_{\text{out}}\times h+b_{\text{out}}$$

where $w_{\text{out}}$ is the weight matrix in the generator output layer, h is the value produced by generator hidden layer and $b_{\text{out}}$ is the bias vector in the generator output layer. While discriminator in GAN also uses a simple Artificial Neural Network (ANN) model that can be represented by equations below.

(4) $$D\left(y\right)=\sigma \left(w_{\text{out}}\times h+b_{\text{out}}\right)$$

(5) $$D\left(\hat{y}\right)=\sigma \left(w_{\text{out}}\times h^{\prime }+b_{\text{out}}\right)$$

σ is activation function, $w_{\text{out}}$ is output layer weight, $b_{\text{out}}$ is output layer bias, and $h^{\prime }$ is transformation results from data produced by the generator. In GAN training, the goal is to optimize the weights and biases in the discriminator so that D(y) close to 1 for the original data and $D(\hat{y})$ approaches 0 for data generated by the generator.

To optimize GAN models, select suitable generator and discriminator architectures based on task complexity and data type. Parameter tuning, like learning rate and latent vector size, is vital for stability and complexity. Utilize Batch Normalization and Residual Connections for training stability. Choose a balanced loss function for the generator and discriminator. Lastly, set batch size and epochs wisely to expedite convergence and minimize overfitting, and ensure optimal GAN model results

### MEMWA control chart based on residual of generative adversarial network model

The Exponentially Weighted Moving Average (EWMA) control chart is a statistical process control (SPC) technique that assigns exponentially decreasing weights to historical observations [17]. To accommodate multivariate processes, the Multivariate EWMA (MEWMA) chart was developed [18]. By considering multiple quality characteristics simultaneously, the MEWMA chart demonstrates enhanced sensitivity to small process shifts, enabling earlier detection of anomalies. Optimal performance of the MEWMA chart is contingent upon the assumption of independent and normally distributed data. While the MEWMA chart exhibits robustness to deviations from normality, its effectiveness can be compromised by autocorrelation in the data.

MEWMA diagrams can be used with GAN models to monitor accuracy by focusing on residuals, the difference between original and GAN-generated data. MEWMA helps detect changes in synthetic data characteristics, providing early alerts for significant deviations. Therefore, the MEWMA equation can be formed as follows [14].

(6) $$M_{t}=\lambda e_{t}+\left(1-\lambda \right)M_{t-1}$$

$M_{t}$ is MEWMA value at t, $e_{t}$ is residual value at t, $M_{t-1}$ is MEWMA value at the previous time, and λ is the weighted exponential where define as 0 < λ < 1 and $M_{0}=0$. Basically, λ could work well while in the interval 0.05≤λ≤0.25, with λ=0.05, λ=0.1, λ=0.2 can be a popular choice. The observation points plotted on the control chart are as follows.

(7) $$\begin{array}{c} T_{t}^{2}=M_{t}^{{}^{\text{'}}}\Sigma_{M_{t}}M_{t} \end{array}$$

where the covariance matrix is

(8) $$\begin{array}{c} \Sigma_{M_{t}}=\frac{\lambda }{\left(2-\lambda \right)}\left[1-{\left(1-\lambda \right)}^{2t}\right]\Sigma \end{array}$$

Observations are said to be out of control when value $T_{t}^{2}>H$, where H is the upper control limit (UCL) which used for MEWMA control chart based on selected lambda (λ) and number of quality characteristics, while the lower control limit (LCL) for MEWMA control charts is equal to 0 because the $T_{t}^{2}$ value is always positive.

### Data source and research variable

This research employed a secondary dataset comprising observational data on clean water production quality. The data was procured from the laboratory of Ngagel II, encompassing the water filtration process from January 1, 2023, to October 24, 2023. The investigation was divided into two phases: Phase I (January 1, 2023 - June 30, 2023) and Phase II (July 1, 2023 - October 24, 2023). Quality assessments were conducted for 296 days post-filtration, a process designed to eliminate fine particles from the previously treated water. Three key water quality parameters were analyzed: pH, turbidity, and organic matter content (measured as KMnO4).

The research variables used in this research are three quality characteristics which are explained in Table below.

**Table 1:** Research Variables.

Quality Characteristics	Description	Unit	Specification
$y_{1}$	pH	–	6.5–7.5
$y_{2}$	Turbidity	NTU	Max 5
$y_{3}$	Organic Substances (KMnO4)	Mg/L	Max 10

### Analysis steps

The research steps analysis taken in this research are as follows.

1. **Problem Definition and Data Collection**: Identify and formalize the specific water quality issues at Surya Sembada water treatment plant Ngagel II, Surabaya. Acquire comprehensive water quality data from January 1, 2023, to October 24, 2023.
2. **Autocorrelation Analysis**: Assess the presence of autocorrelation in the water quality time series using the Multivariate Cross-Correlation Function (MCCF) plot. Identify significant autocorrelation lags to inform subsequent modeling decisions.
3. **Data Partitioning**: Divide the dataset into training data (phase I: January 1, 2023, to June 30, 2023) and testing data (phase II: July 1, 2023, to October 24, 2023).
4. **GAN Modeling**: Develop a Generative Adversarial Network (GAN) model to generate synthetic water quality data. Optimize GAN hyperparameters (layers, epochs, batch size) on the training set to achieve optimal performance. Apply the trained GAN model to generate synthetic data for the testing set.
5. **Residual Analysis**: Calculate residuals between the actual and synthetic water quality data for the training set. Assess the normality of residuals using the Shapiro-Wilk test and the presence of autocorrelation using the MCCF plot.
6. **Multivariate EWMA Control Chart**: Develop a Multivariate Exponentially Weighted Moving Average (MEWMA) control chart using the residuals from the training set. Establish control limits based on in-control assumptions. Monitor the testing set residuals using the developed control chart to detect abnormal patterns or out-of-control signals.
7. **Conclusion and Recommendations**: Findings and Implications: Summarize the key findings of the study, including the performance of the GAN model, the effectiveness of the control chart in detecting anomalies, and the identified root causes of out-of-control signals. Provide actionable recommendations for im-proving water quality management at Surya Sembada water treatment plant Ngagel II, Surabaya. This may involve process adjustments, data-driven decision making, or implementation of advanced monitoring systems.

**Pseudocode:**

**Table:** 

**Input:**
X_t ← multivariate time series data for *t* = 1 to T
λ ← smoothing parameter for MEWMA
Phase_I_range, Phase_II_range ← time ranges for training and monitoring
**Output:**
Out-of-control signals from MEWMA chart
**Procedure:**
**Step 1:** Check Autocorrelation
If MCCF(X_t) shows autocorrelation:
Proceed to GAN modeling
**Step 2:** Train GAN on Phase_I
Initialize Generator G and Discriminator D
For epoch = 1 to N:
Sample real data batch from Phase_I
Generate fake samples: X_fake ← G(noise)
Train D on real and fake data
Train G to fool D (update via loss minimization)
End For
**Step 3:** Generate Residuals:
For each t in Phase_I and Phase_II:
X_pred[t] ← G(noise or input features at t)
Residual[t] ← X_actual[t] - X_pred[t]
**Step 4:** Validate Residuals
If MCCF(Residual) indicates autocorrelation is removed:
Proceed
Else:
Tune GAN and retrain
**Step 5:** Build MEWMA Control Chart Phase_I
Initialize Z_0 ← 0
For t in Phase_I:
Z_t ← λ * Residual[t] + (1 - λ) * Z_{t-1}
T2[t] ← Z_tᵀ * Σ_Z⁻¹ * Z_t
Compute UCL
Flag any T2[t] > UCL as out-of-control
Clean the out-of-control signal
**Step 6:** Monitor with MEWMA Phase_II
For t in Phase_II:
Z_t ← λ * Residual[t] + (1 - λ) * Z_{t-1}
T2[t] ← Z_tᵀ * Σ_Z⁻¹ * Z_t
If T2[t] > UCL:
Signal out-of-control at time t
**End Procedure**

## Method validation

### Autocorrelation analysis

To use control charts for analysis, observation data must be independent. If autocorrelation is detected, it must be addressed. Prior to autocorrelation checking, the three quality characteristics are standardized due to their different units. Autocorrelation is checked using the MCCF plot for each quality characteristic of water, as shown in Table 2.

**Table 2:** MCCF Result of Quality Characteristics.

Variable/Lag	0	1	2	3	4	5	6	7	8	9
pH	++-	++.	++.	++.	++.	++.	++.	++.	++.	++.
Turbidity	+++	+++	+++	+++	+++	++.	++.	++.	++.	++.
KMnO4	-++	..+	-.+	-.+	-.+	..+	..+	..+	..+	…
**Variable / Lag**	**10**	**11**	**12**	**13**	**14**	**15**	**16**	**17**	**18**	**19**
pH	++.	++.	++.	++.	++.	++.	++.	++.	++.	++.
Turbidity	++.	++.	++.	++.	++.	++.	++.	++.	++.	++.
KMnO4	…	…	…	…	…	…	…	…	…	…

Table 2 reveals a pronounced and persistent positive autocorrelation for pH and turbidity across nearly all lags, suggesting a strong temporal dependency in these parameters. This indicates that fluctuations in pH and turbidity tend to be correlated with their preceding values over extended periods. In contrast, KMnO4 exhibits a more erratic and inconsistent autocorrelation pattern, characterized by substantial fluctuations and a lack of discernible regularity. Collectively, these findings imply a higher degree of stationarity for pH and turbidity compared to KMnO4. The presence of autocorrelation in the clean water process data is attributable to the continuous nature of the production process. To address the challenges posed by autocorrelation in the observational data, the application of Generative Adversarial Network (GAN) modeling is proposed.

Based on Table 3, the Generative Adversarial Network (GAN) employed in this study consists of two main components: a Generator (G) and a Discriminator (D), both implemented using Artificial Neural Networks (ANNs).

**Table 3:** Architecture & hyper-parameter.

Component	Hyper-Parameter
Generator Layer 1	Number of Neuron = 200
	Activation = LeakyReLu (Alpha 0.01)
Generator Layer 2	Number of Neuron = 600
	Activation = LeakyReLu (Alpha 0.01)
Generator Layer 3	Number of Neuron = 3
	Activation = Linear
Discriminator Layer 1	Number of Neuron = 600
	Activation = LeakyReLu (Alpha 0.01)
Discriminator Layer 2	Number of Neuron = 200
	Activation = LeakyReLu (Alpha 0.01)
Discriminator Layer 3	Number of Neuron = 1
	Activation = Sigmoid
Discriminator Compile	Loss Function = Binary Cross Entropy Optimizer = Adam (Lr 0.0001, Beta 0.3)
Compile of Model	Optimizer = Adam (Learning Rate = 0.0001, Beta = 0.3)

Generator (G): The generator's role is to produce synthetic data that closely resembles the real water quality data. It takes an input vector, which can be random noise or actual historical data, and transforms it through multiple layers to generate predicted water quality values. The specific architecture of our generator is as follows:

Layer 1: A dense layer with 200 neurons and a Leaky ReLU activation function (Alpha 0.01).

Layer 2: A dense layer with 600 neurons and a Leaky ReLU activation function (Alpha 0.01).

Layer 3 (Output Layer): A dense layer with 3 neurons (corresponding to pH, turbidity, and KMnO4) and a linear activation function, as represented by the equation G(yt​)=σ(hout​) where hout​=wout​×*h*+bout​. Here, wout​ is the weight matrix, h is the output from the hidden layer, and bout​ is the bias vector.

Discriminator (D): The discriminator's function is to distinguish between the real water quality data and the synthetic data generated by the generator. It also employs an ANN structure:

Layer 1: A dense layer with 600 neurons and a Leaky ReLU activation function (Alpha 0.01).

Layer 2: A dense layer with 200 neurons and a Leaky ReLU activation function (Alpha 0.01).

Layer 3 (Output Layer): A dense layer with 1 neuron and a Sigmoid activation function, represented by D(y).

The selection of the Leaky Rectified Linear Unit (Leaky ReLU) activation function, with an alpha parameter of 0.01, is a strategic choice for enhancing the stability of GAN architectures. This function is designed to mitigate the "dying ReLU" problem, a common issue in neural network training where neurons can become permanently inactive. By allowing a small, non-zero gradient for negative inputs, Leaky ReLU ensures a continuous gradient flow, which is critical for the robust and stable training of both the generator and discriminator networks.

The GAN training process involves an iterative improvement where the generator aims to create more realistic data, and the discriminator strives to become better at distinguishing real from fake data. The discriminator is optimized to yield a value close to 1 for original data and close to 0 for generated data. Hyperparameter tuning was a critical step to optimize the GAN model's performance, particularly in minimizing model residuals. The Adam optimizer was used for both the discriminator and the overall model compilation, with a learning rate of 0.0001 and a Beta parameter of 0.3.

### Generative adversarial networks’ modelling

GAN-based ANN is employed to address autocorrelation in clean water production. Optimal GAN architecture and hyperparameters are determined through hyperparameter tuning to minimize model residuals. These optimized parameters will be applied to the modeling process in phase II.

**1. Phase I GAN’s Modelling**

Phase I of the GAN modeling process utilized clean water data spanning January 1 to June 30, 2023. The network architecture and hyperparameters employed are detailed in Table 3. Model training was conducted using the Adam optimizer with a learning rate of 0.0001 and parameter β, as outlined in Table 6. A range of epochs were explored during training, and resulting loss values for each epoch are tabulated in Table 4. Training metrics were captured and stored within a history variable for subsequent analysis.

**Table 4:** Loss values for generator and discriminator.

Epoch	Generator Loss	Discriminator Loss
600	0.3751	0.9538
650	0.3748	0.9467
700	0.3729	0.9443

To optimize GAN model performance, a hyperparameter tuning process was conducted, focusing on the epoch value. From three candidate epochs, the model trained for 700 epochs exhibited the lowest loss values, suggesting superior convergence and predictive accuracy. This outcome indicates the generator's effectiveness in producing highly realistic synthetic data, thereby challenging the discriminator's ability to distinguish between genuine and artificial samples. Model evaluation was conducted using Mean Squared Error (MSE), Root Mean Squared Error (RMSE), and Mean Absolute Error (MAE) metrics. These metrics quantified the discrepancy between predicted and actual values for the three quality characteristics generated by the GAN model. The resulting evaluation results are summarized in Table 5.

**Table 5:** Evaluation metrics.

Epoch	$\bar{\text{MSE}}$	$\bar{\text{RMSE}}$	$\bar{\text{MAE}}$
600	0.0067	0.0822	0.0633
**650**	**0.0054**	**0.0738**	**0.0556**
700	0.0071	0.0846	0.0637

**Fig. 2:**
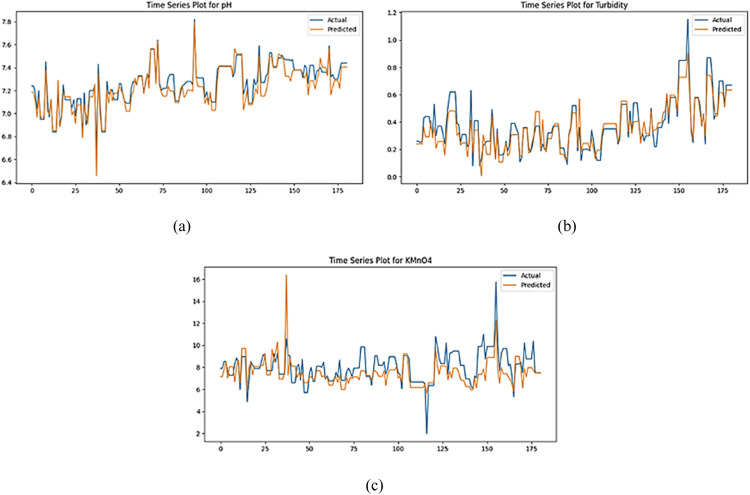
Time series plot of actual data and predicted data in phase I.

The GAN model trained for 650 epochs exhibited the most promising performance, demonstrating superior predictive accuracy. Specifically, this configuration achieved an MSE of 0.0054, RMSE of 0.0738, and MAE of 0.0556. These performance metrics were collectively the lowest among all evaluated epoch candidates. Consequently, the 650 epoch configuration was selected as it most effectively fulfilled the primary objective of the Phase I modeling, which was to minimize model residuals and thereby enhance the model's predictive capabilities. As visualized in Fig. 2, a comparison of predicted and actual values provides empirical evidence supporting the model's reliability.

Figs. 2(a) and 12(b) show that the data pattern of the predicted values for the quality characteristics of pH and turbidity from GAN modeling has a pattern that is similar or follows the actual data values for phase I. Meanwhile, the quality charac-teristics of KMnO4 can be seen in Fig. 2(c) has a predicted data pattern that tends to be on average or in the middle with actual data. This proves that predictions by modeling using GANs can be used because the patterns produced from predicted data tend to have the same patterns as actual data.\

**2. Phase II GAN’s Modelling**

**Fig. 3:**
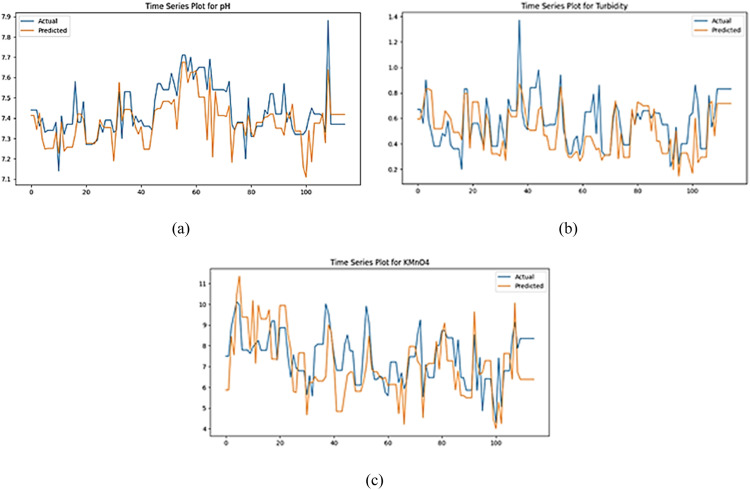
Time series plot of actual data and predicted data in phase II.

Phase II GAN modeling was conducted using identical architectural and hyperparameter settings as employed in phase I. The model was trained on clean water data spanning from July 1 to October 24, 2023. Subsequent to training, predicted values were generated and compared to corresponding actual data points, as visualized in Fig. 3. An analysis of Fig. 3 reveals that the predicted values for all three quality characteristics exhibit a pattern that deviates significantly from the observed trends in the phase II actual data. Nevertheless, the predicted data points are distributed within a range proximate to the mean of the actual data. These findings suggest that the GAN model’s predictive capabilities during phase II were suboptimal in capturing the specific nuances and fluctuations of the actual data. Consequently, the generated data displayed markedly different patterns compared to the ground truth. To quantitatively assess these pattern discrepancies, a control chart analysis will be performed.

### Control chart assumption testing

The residuals of the GAN-modeled water quality data from Phase I observations are assessed for autocorrelation using MCCF plots prior to multivariate normality testing, a prerequisite for control chart implementation.

Analysis of Table 6 indicates that GAN modeling effectively mitigates autocorrelation present in Phase I observational data for the examined quality characteristics. The pH variable exhibited pronounced cyclical patterns, evidenced by significant and strong autocorrelation at initial and multiple higher lags. Turbidity data displayed higher variability and a more random structure, although significant autocorrelation was detected at specific lags. KMnO4 demonstrated strong autocorrelation at early lags, transitioning to a more stable pattern at subsequent lags. Subsequent to autocorrelation assessments of GAN model residuals, a Shapiro-Wilk test was employed to evaluate multivariate normality. Results are tabulated in Table 7.

**Table 6:** MCCF result based on GAN Phase I residuals.

Variable / Lag	0	1	2	3	4	5	6	7	8	9
pH	+-+	+-+	+..	…	…	…	…	…	…	…
Turbidity	-+.	.+.	.+.	…	…	…	..+	…	…	…
KMnO4	+.+	+.+	…	…	…	…	…	…	…	…
**Variable / Lag**	**10**	**11**	**12**	**13**	**14**	**15**	**16**	**17**	**18**	**19**
pH	…	.-.	…	..+	..+	…	…	…	…	…
Turbidity	…	…	..-	..-	-+-	.+.	…	…	…	…
KMnO4	…	+..	…	…	…	…	…	…	…	…

**Table 7:** Normal multivariate test for residuals phase I.

Shapiro Wilk	P-Value	Decision
0.86278	< 2.2e-16	Not normally distributed

Based on the multivariate normal distribution test reveals that the clean water quality data doesn’t follow a multivariate normal distribution, as the p-value is less than the 5 % alpha level. In this study, we made a deliberate methodological decision not to apply transformations, such as Box-Cox, to the GAN-generated residuals. The primary reason for this approach was to analyze the original pattern of the process as it was formed from the modeling results. We believe that while transformation could help in satisfying the normality assumption, it would also alter the inherent data patterns. This alteration could potentially mask or delay the detection of anomalies, which would be counterproductive to the primary objective of a control chart, which is rapid process monitoring. This decision was further supported by the established robustness of the MEWMA chart to violations of the normality assumption.

### MEWMA control chart based on residuals of GAN model

**Fig. 4:**
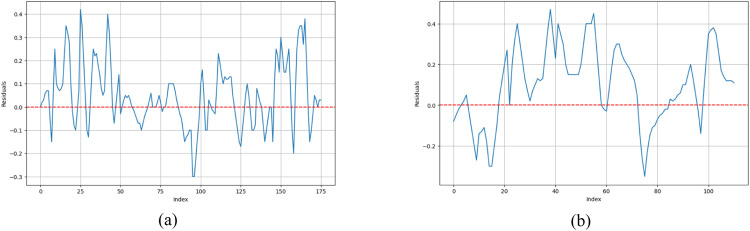
Residual Plot: (a) Phase 1, (b) Phase 2.

In this part, the residual from the GAN best model is used to form the Multivariate Exponentially Weighted Moving Average (MEWMA) control chart to monitor multiple quality characteristics simultaneously. MEWMA excels at detecting small process shifts and is sensitive to changes in the process mean. Control limits are determined based on Average Run Length (ARL) criteria, with a target ARL of 370 achieved through appropriate weighting (λ). The determination of the Upper Control Limit (UCL) for the MEWMA control chart is generally obtained through extensive Monte Carlo simulations, see detailed explanation in [14]. Residuals $e_{t}$​ are defined as the difference between the actual observed water quality data $y_{t}$ and the synthetic or predicted data ${\hat{y}}_{t}\;$generated by the best GAN model. The calculation of residuals is straightforward: $e_{t}=y_{t}-{\hat{y}}_{t}$. These residuals represent the unexplained variation in the water quality data after accounting for the patterns captured by the GAN model. Fig. 4 visually presents these residuals over time for the two distinct study periods: (a) Phase I, which will be used for control chart calibration, and (b) Phase II, which will be used for prospective monitoring. The key objective now is to determine if this residual process is stable or if it exhibits any non-random behavior using the MEWMA chart.

**1. MEWMA Control Chart Based on Residual of GAN Models Phase I**

**Fig. 5:**
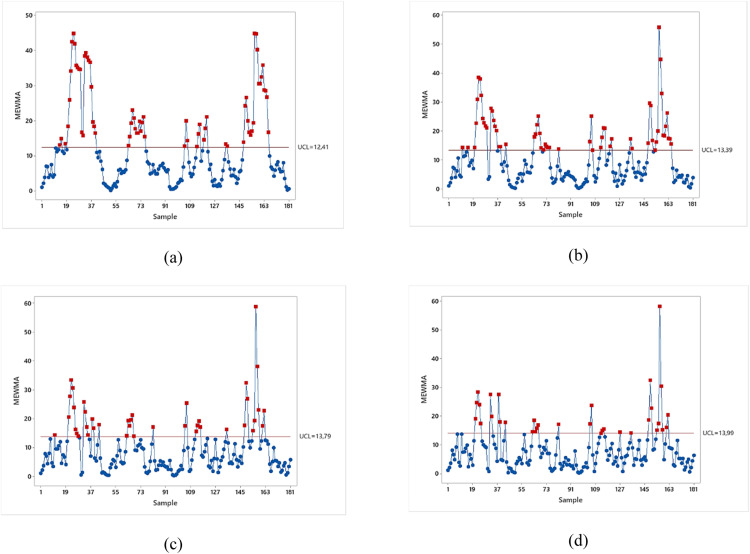
MEWMA control chart based on residual of GAN Phase I: (a) λ = 0.1, (b) λ = 0.2, (c) λ = 0.3, (d) λ = 0.4.

Fig. 5 shows the monitoring results of the MEWMA Control Chart based on the residual of GAN Phase I for several values of λ. The summary of the number of out-of-control signals is presented in Table 8. It was observed that smaller λ values, such as 0.1, 0.2, and 0.3, resulted in an excessive number of out-of-control (OOC) signals. This indicated a potential oversensitivity, where the chart was reacting to inherent process noise rather than significant, actionable shifts. From an operational perspective, such oversensitivity is undesirable as it can lead to frequent false alarms and costly, unnecessary process interventions. In contrast, the MEWMA chart with λ = 0.4 provided a more stable and realistic view of the process, identifying 33 out-of-control points that could be meaningfully investigated. This value was chosen because it was sensitive enough to detect important deviations without being oversensitive.

**Table 8:** Number of out of out-of-control signal for MEWMA chart based on residual of GAN Phase I.

Smoothing Parameter (λ)	Number of Out of Control	UCL
0.1	63	12.41
0.2	57	13.39
0.3	41	13.79
0.4	33	13.99

The selection of λ = 0.4 for the MEWMA chart was a deliberate choice reflecting a trade-off between sensitivity and operational practicality. As detailed in Table 8, smaller smoothing parameters, such as 0.1, 0.2, and 0.3, were considered overly sensitive, yielding an excessive number of out-of-control signals. From an operational perspective, such oversensitivity is undesirable as it leads to frequent false alarms and costly, unnecessary process interventions. In contrast, λ = 0.4 provided a more stable and realistic view by identifying 33 out-of-control points that were suitable for meaningful investigation. This value was ultimately chosen because it was sensitive enough to detect important deviations without being oversensitive to normal process noise, thereby establishing the most balanced and actionable monitoring framework. Therefore, the selection of λ = 0.4 was based on a visual and practical assessment of the Phase I results to achieve the most balanced and actionable monitoring framework for this specific case.

**Fig. 6:**
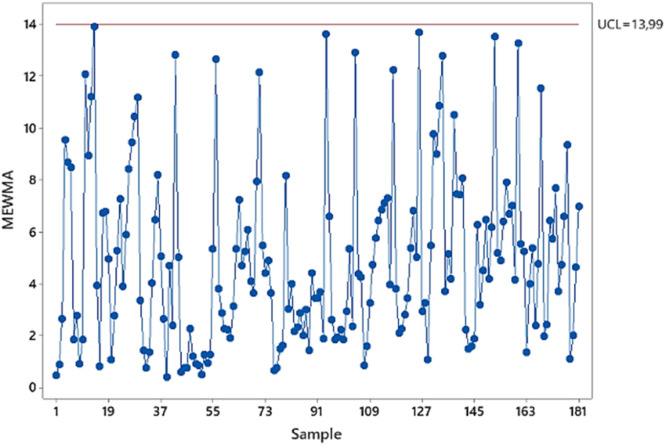
MEWMA control chart based on residual of GAN phase I (In Control).

Fig. 6 depicts an updated MEWMA control chart for Phase I GAN model residuals (UCL=13.99, λ=0.4). No out-of-control signals were detected post-intervention (removal of the highest outlier). The Phase I water production GAN model is now statistically in-control and prepared for Phase II monitoring.

**2. MEWMA Control Chart Based on Residual of GAN Models Phase II**

**Fig. 7:**
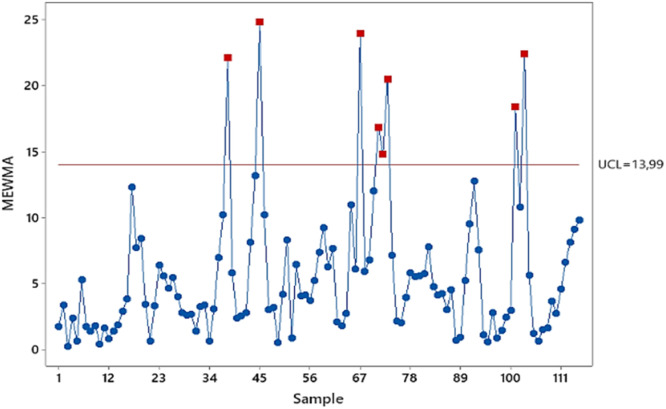
MEWMA control chart based on residual of GAN phase II.

Fig. 7 depicts a Phase II Multivariate Exponentially Weighted Moving Average (MEWMA) control chart with λ = 0.4 and UCL = 13.99. The presence of eight data points exceeding the upper control limit indicates a statistically significant deviation from the process mean in clean water production. This suggests the process is out of control. To identify the specific factors contributing to this process shift, a detailed analysis of residuals from the Phase I Generative Adversarial Network (GAN) model is warranted. These residuals, representing the discrepancies between observed and predicted clean water quality characteristics, can provide valuable insights into the underlying process dynamics. By examining combinations of these residual patterns, it is possible to pinpoint potential root causes of the process excursion. Such knowledge is essential for implementing corrective actions to restore the process to a state of statistical control and ensure the consistent delivery of high-quality clean water to customers.

This research introduces a novel approach to water quality monitoring by integrating Generative Adversarial Network (GAN) residuals into a Multivariate Exponentially Weighted Moving Average (MEWMA) control chart. Analysis of Surya Sembada's clean water production process revealed that current pH, turbidity, and KMnO4 levels are within specified limits, indicative of a controlled process. The proposed GAN model effectively mitigated autocorrelation in residuals and exhibited superior predictive performance, as quantified by lower MSE, RMSE, and MAE compared to alternative methods. While the MEWMA chart effectively detected process deviations in Phase II, the computational demands of GAN training underscore the need for careful consideration of computational efficiency and robustness to process shifts for practical applications. Future research should explore strategies to optimize computational performance and enhance the model's sensitivity to subtle process variations. Also, some robust approaches can be applied to the control chart.

### Limitations

This research relied on a secondary dataset obtained from the Ngagel II Surya Sembada laboratory, which introduces several limitations:

1. **Restricted Time Coverage**
  The dataset spans only a 10-month period (January 1 to October 24, 2023), which may not capture long-term seasonal variations or periodic trends affecting water quality, such as rainfall patterns or operational shifts across years.
2. **Limited Parameter Scope**
  Only three parameters—pH, turbidity, and KMnO₄ (organic matter content)—were analyzed. Other potentially influential indicators such as temperature, dissolved oxygen, chlorine residuals, or microbial content were not included, potentially limiting the comprehensiveness of the water quality assessment.
3. **Single-Source & Location Dependency**
  All data were sourced from a single water treatment plant (Ngagel II), potentially affecting generalizability. The results may not be directly applicable to other plants with different water sources, treatment technologies, or environmental conditions.
4. **Secondary Data Constraints**
  As the data were not collected primarily for this study, control over measurement methods, sampling intervals, and potential data inconsistencies is limited. Any errors or biases in original data recording may propagate through the analysis.
5. **Model Performance Degradation and Process Non-Stationarity**
  A significant limitation observed was the degradation of the Generative Adversarial Network (GAN) model's predictive performance when the model trained on Phase I data was applied to the Phase II dataset. The model's predictions deviated significantly from the observed trends in Phase II, indicating they were suboptimal in capturing the new data's specific nuances and fluctuations. This performance decline suggests that the underlying water quality process may be non-stationary, with its statistical properties shifting between the two time periods. The static nature of the trained model meant it could not adapt to this process drift, which tempers expectations for the long-term deployment of such models and underscores the need for periodic retraining or adaptive learning frameworks to maintain effectiveness.

Based on Table 9, for future research, a comprehensive comparative analysis will be undertaken to rigorously assess the performance of the GAN-MEWMA methodology against other established and emerging machine learning methods, such as Long Short-Term Memory (LSTM) networks, XGBoost, and Vector Autoregression (VAR) models. This systematic evaluation will focus on key metrics, including predictive accuracy (e.g., lower MSE, RMSE, MAE for residuals), efficacy in autocorrelation reduction, computational efficiency, and robustness in detecting process shifts, thereby more definitively positioning the relative advantages and limitations of the proposed GAN-MEWMA framework within the broader landscape of statistical process control.

**Table 9:** Comparison of Characteristics for several models.

Model	Characteristics
GAN	• Known for generating realistic synthetic data through a competitive process between a generator and a discriminator. • Can be used to overcome data limitations in forecasting applications. • The training process can have significant computational considerations.
LSTM	• An advanced machine learning algorithm suitable for time series analysis and water quality forecasting. • Can be used in hybrid models for multivariate water quality forecasting.
XGBoost	• An advanced machine learning algorithm that can be employed for tasks like water quality classification.
VAR	• A traditional time series method used for forecasting multivariate data, such as water quality.

To improve upon the current methodology, future work could focus on incorporating more advanced and systematic approaches to model tuning. While the hyperparameter tuning process used in this study successfully identified an effective model by comparing a few candidate epoch values to minimize residuals, a more rigorous process could yield further improvements. Therefore, it is recommended that future research explores the use of formal and automated hyperparameter optimization techniques. Implementing methods such as Grid Search, Random Search, or Bayesian Optimization could lead to a more transparent and reproducible model selection process.

## CRediT authorship contribution statement

**Muhammad Ahsan:** Validation, Visualization, Writing – original draft, Conceptualization. **Raditya Widi Indarsanto:** Validation, Visualization, Writing – original draft, Conceptualization. **Kevin Agung Fernanda Rifki:** Writing – original draft, Conceptualization. **Muhammad Hisyam Lee:** Writing – review & editing.

## Declaration of interests

The authors declare that they have no known competing financial interests or personal relationships that could have appeared to influence the work reported in this paper.

## Data Availability

Data will be made available on request.
